# An Open-Source Package for Thermal and Multispectral Image Analysis for Plants in Glasshouse

**DOI:** 10.3390/plants12020317

**Published:** 2023-01-09

**Authors:** Neelesh Sharma, Bikram Pratap Banerjee, Matthew Hayden, Surya Kant

**Affiliations:** 1 Agriculture Victoria, Grains Innovation Park, 110 Natimuk Rd, Horsham, VIC 3400, Australia; 2 AgriBio, Centre for AgriBioscience, Agriculture Victoria, 5 Ring Road, Melbourne, VIC 3083, Australia; 3 School of Applied Systems Biology, La Trobe University, Melbourne, VIC 3083, Australia

**Keywords:** thermal, multispectral, image processing, co-registration, illumination correction, segmentation

## Abstract

Advanced plant phenotyping techniques to measure biophysical traits of crops are helping to deliver improved crop varieties faster. Phenotyping of plants using different sensors for image acquisition and its analysis with novel computational algorithms are increasingly being adapted to measure plant traits. Thermal and multispectral imagery provides novel opportunities to reliably phenotype crop genotypes tested for biotic and abiotic stresses under glasshouse conditions. However, optimization for image acquisition, pre-processing, and analysis is required to correct for optical distortion, image co-registration, radiometric rescaling, and illumination correction. This study provides a computational pipeline that optimizes these issues and synchronizes image acquisition from thermal and multispectral sensors. The image processing pipeline provides a processed stacked image comprising RGB, green, red, NIR, red edge, and thermal, containing only the pixels present in the object of interest, e.g., plant canopy. These multimodal outputs in thermal and multispectral imageries of the plants can be compared and analysed mutually to provide complementary insights and develop vegetative indices effectively. This study offers digital platform and analytics to monitor early symptoms of biotic and abiotic stresses and to screen a large number of genotypes for improved growth and productivity. The pipeline is packaged as open source and is hosted online so that it can be utilized by researchers working with similar sensors for crop phenotyping.

## 1. Introduction

Plant phenotyping characterises growth and biophysical traits at different plant development stages [1]. Conventional phenotyping methods (e.g., visual observations and destructive sampling) are prone to operator bias, time-consuming, and often destructive [2,3]. Image-based high-throughput plant phenotyping is a promising alternative to conventional phenotyping and has been widely used to measure plant morphological and agronomical traits. Imaging systems, including thermal and multispectral sensors, provide a non-invasive and non-destructive method for detecting emitted and reflected electromagnetic radiation from plant canopies to study plant traits such as growth, biomass accumulation, and stress symptoms [4,5].

Optical images can be used to measure different plant traits, including leaf area, height, canopy biomass, and yield [6]. Multispectral imaging is widely used to extract detailed information about crop attributes by capturing spectral data cubes, consisting of two-dimensional images under different wavelengths [7]. Mathematical combinations of spectral wavebands are often used to create vegetative indices (VIs), such as Normalized Difference Vegetation Index (NDVI) [8] and Enhanced Vegetation Index (EVI) [9], which are used to estimate green biomass [10,11]. Similarly, Normalized Difference Red-Edge (NDRE) [12] and Red-edge Chlorophyll Index (RCI) [13,14] are used to predict chlorophyll concentration in plant tissues [15]. Multispectral imaging processing and machine learning techniques have also been used to detect abiotic and biotic stresses in plants, such as root water stress [16,17], and tomato spotted wilt virus and powdery mildew [18].

Thermal imaging can be used to monitor subtle changes in the temperature of plant canopies over different plant growth stages and in response to environmental conditions. Unlike multispectral imaging, thermal imaging measures the emitted radiation from plants, thereby not requiring an illumination source [19]; hence, thermal sensing can be easily employed at night to observe diurnal changes in plants, thereby enabling study of critical plant physiological processes such as diurnal water loss due to transpiration [20,21]. Moreover, thermal images can detect subtle variations in plant temperature due to water stress. The temperature of plants has been shown to increase long before the appearance of chlorotic or necrotic patches in response to disease. Currently, thermal imagery is widely used for irrigation scheduling [22], detecting stress in canopies due to pathogens [23], heat stress, and stomatal conductance [24,25].

Vieira and Ferrarezi [26] used a handheld thermal camera to determine water stress and assess the water potential of citrus plants growing under glasshouse conditions. In another study by Hu, et al. [27], thermal imaging was combined with a back propagation neural network to compare predictions of Infrared Crop Water Stress Index (ICWSI) with yield. Grant, Chaves and Jones [24] applied thermal imaging under a controlled environment to study the reaction of plants (grapevines, beans, and lupins) under irrigated and non-irrigated conditions. The study observed a significant correlation between temperature and stomatal conductance; however, it also highlighted potential limitations of thermal imaging, such as inaccuracy in temperature values, time-consuming data analysis, and a lack of reliable references to calibrate temperature.

Thermal imaging paired with multispectral imaging provides a dimensional modality to study the physiological response of plants to stress [1]. For instance, thermal images can detect subtle temperature changes, while multispectral images provide complementary information on the presence of any biotic stress (observed from colour change), and crop biomass [28,29,30]. Further, specific technical constraints of unimodal datasets can be resolved through multimodal data fusion. For example, thermal imaging is prone to the temperature of in-scene background targets when measuring plant temperature. The fusion of thermal and spectral imaging enables segmentation algorithms to mask background thermal noise and extract pure thermal pixels from plants [1]. Leinonen and Jones [25] combined images from thermal, red, and NIR bands, which were utilised to separate the plants from the background soil. Their study suggested that the co-registration of visible and thermal images followed by the classification of pixels in the visible spectrum is essential for accurately profiling canopy temperature. Stutsel, et al. [31] used thermal cameras to see variation in temperature of tomato plants under salinity stress. Pixel information from the green–red vegetation index was derived from RGB images and was used to outline individual plants from the soil pixel. Bai, et al. [32] calculated Crop Water Stress Index (CWSI) and Growth Index (GI) using an image processing pipeline created for thermal and multispectral images. The study advocated that fusion of thermal and multispectral imaging in glasshouse conditions has potential to efficiently phenotype wheat genotypes for drought tolerance. Cucho-Padin, et al. [33] have fused the IR and RGB images from a thermal camera to develop software to calculate the Crop Water Stress Index (CWSI) and Green–Red Vegetative Index (GRVI). Bulanon, et al. [34] fused thermal and visible images for orange fruit detection using Laplacian pyramid transform and fuzzy logic. This study highlighted the benefits image fusion compared to simply using the thermal images for improved efficacy of fruit detection. Despite the availability of a few studies involving the fusion of thermal imaging with other visible imaging in agriculture applications, its wide scale adoption is still limited. To large extent, this is due to the unavailability of an image processing tool to enable ease of data analytics.

Thermal and multispectral imaging are used in both aerial and handheld modes to study plant growth and response to treatments under field and glasshouses conditions [35]. Aerial imageries are beneficial for covering large areas in fields. Handheld sensors are advantageous for studying individual plants, as the analysis of different sections of the canopy can be performed with higher spatial resolution and accuracy [36]. However, both multispectral and thermal imaging are impacted by various environmental factors (including air temperature, humidity, haze, illumination intensity and direction) and, therefore, require correction of raw images before further analysis [4,37]. This study aimed to (i) develop an image processing pipeline to optimise thermal and multispectral imagery under glasshouse conditions; (ii) reduce the processing time by incorporating batch processing routines including image calibration, registration, illumination adjustment, temperature rescaling, segmentation, extraction of vegetation indices, and temperature profiles; and (iii) demonstrate the efficacy of the developed package to detect early symptoms of heat stress in wheat plants.

## 2. Materials and Methods

### 2.1. Experiment Setup

The experiment was conducted in a glasshouse at the Grains Innovation Park, Horsham, Victoria, Australia. Wheat plants were grown in pots to test and optimise thermal and multispectral imagery. The growing conditions in the glasshouse were 24 °C and 15 °C during day and night, respectively, with relative humidity ranging 55–65%. The imaging was done under natural light conditions.

### 2.2. Integrated Sensor Platform and Imaging Setups

The experiment used a thermal (FLIR T640, Teledyne FLIR LLC, Wilsonville, OR, USA) and a multispectral (Parrot Sequoia, Parrot SA, Paris, France) sensor to capture multimodal data. The FLIR T640 sensor provides a resolution of 640 × 480 pixels and can detect temperature ranges from −40 to 2000 °C. The camera provides a precision of ±2 °C and thermal sensitivity of less than 0.03 °C at 30 °C. The Parrot Sequoia was used to capture multispectral images with four spectral channels, including green, red, infrared (IR), and red-edge bands with a resolution of 1290 × 960 pixels at 12 Mpix. The central wavelength and wavelength width of the channels are green: 550 ± 40 nm, red: 660 ± 40 nm, red-edge: 735 ± 10 nm and NIR: 790 ± 40 nm [38]. Additionally, the multispectral sensor captures a standard red–green–blue (RGB) colour image with a resolution of 4608 × 3695 pixels.

**Figure 1:**
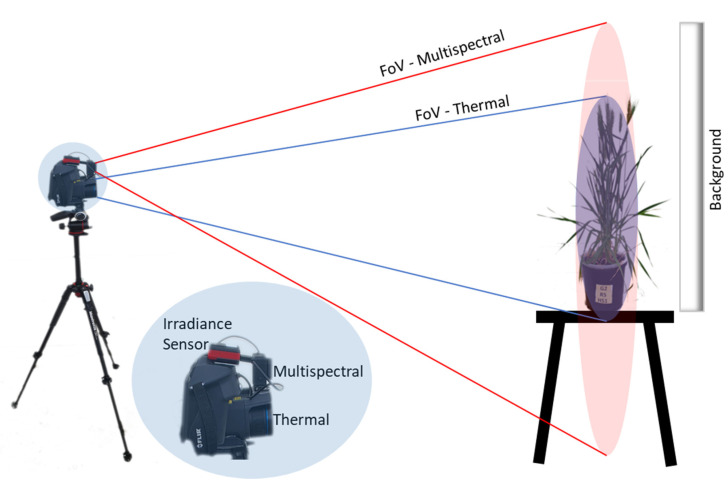
Integrated multimodal and imaging setup. A multispectral camera was physically mounted on the top of a thermal camera using a magnetic mount assembly to provide a uniform field-of-view (FoV). The irradiance sensor was mounted on the top. During imaging, a radiometric calibration target with 80 percent reflectivity was placed behind the plants.

A special arrangement was required to pair the thermal and multispectral sensors and provide a systematic overlap of respective field-of-views (FoVs) and pixel-to-pixel matching between the sensors. The thermal and multispectral cameras were integrated using a magnetic mount assembly (Figure 1). The physical pairing of the two sensors ensured a fixed relative orientation, with the multispectral sensor providing a larger FoV to envelop the thermal sensor’s FoV. The irradiance sensor of the multispectral camera was positioned over the thermal camera to capture variations in local illumination levels during imaging. Finally, a white background target with 80 percent reflectivity was placed behind the object plane to act as a radiometric calibration target. This provided a spectral contrast between the plant and background, which helped to digitally extract the plant and avoid background noise. The camera setup was kept stationary, and the image acquisition was triggered after manually placing a potted plant in front of the imaging setup.

### 2.3. Image Processing

**Figure 2:**
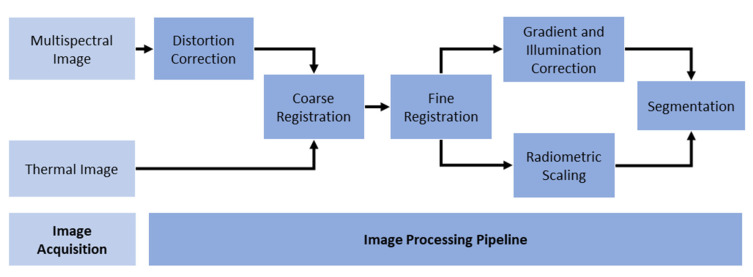
The image processing pipeline. The steps include image acquisition by thermal and multispectral cameras, and image processing for distortion correction of multispectral images, image registration (coarse and fine), radiometric scaling of thermal images, and illumination correction of multispectral images.

A processing pipeline was developed to correct geometric distortions, image-to-image registration, radiometric/illumination correction, and segmentation of captured images (Figure 2). The image processing pipeline aimed to correct irregularities in the images due to intrinsic factors (e.g., camera distortion), ambient factors (e.g., light and temperature variations), and segment areas of interest (i.e., the plant canopies from the background). The pipeline also aimed to process images in a batch to simplify the processing. All codes were written in MATLAB to produce a library package which is available at https://github.com/SmartSense-iHub/Thermal-and-Multispectral-Image-Analysis-Processing-Pipeline.git (accessed on 12 November 2022). A MATLAB library Natural-Order Filename Sort (Nsortfiles) [39] was used to load thermal and multispectral images in sequential order.

#### 2.3.1. Correction of Radial Optical Distortions in Multispectral Images

**Figure 3:**
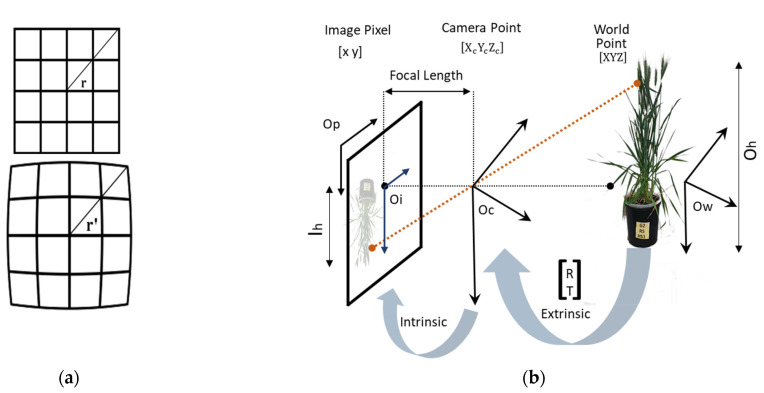
Process of capturing images and radial optical distortion. (**a**) An image without distortion (top) and image with radial barrel distortion (bottom) where r is the off-axis image distance, which increases with distortion. (**b**) Extrinsic parameters (Rotation (R) and Translation (T)) are used to convert the 3D world plane coordinates ($O_{w}$) to a 3D camera plane coordinates ($O_{c}$), which are converted to 2D image coordinates ($O_{i}$) with the help of intrinsic parameters; $O_{p}$ represents the pixel plane.

Multispectral sensors have significant radial barrel distortion and different spatial coverage among bands that lead to misregistration effects [40]. Radial distortion occurs when the light rays bend more towards the edges of a lens than at its optical centre, and it is inversely proportional to the size of the lens. Radial distortion occurs if the FoV of a fore−optics lens is greater than the size of the image sensor that captures the image. The inward or outward displacement of light rays before hitting the sensor from its ideal location causes the straight lines on the image to render the shapes of an arc due to radial distortion [41,42]. Radial distortion is the measure of the image height (I_h_) divided by object height (O_h_), i.e., transverse magnification (M = $\frac{I_{h}}{O_{h}}$) with the off-axis image distance (r). An increase in *M* with r results in pincushion distortion, whereas barrel distortion is observed when *M* decreases with r [41] (Figure 3a).

Camera calibration involves determining the intrinsic, extrinsic, and distortion parameters. Extrinsic parameters transfer the 3D world coordinates [X Y Z] to the 3D camera coordinates [$X_{c}$ $Y_{c}$ $Z_{c}$] [43]. The extrinsic parameters consist of Rotation (R) and Translation (T) (Figure 3b).

Three intrinsic parameters—principal points (optical centre), focal length, and skew coefficient(s) convert 3D camera coordinates to 2D pixel coordinates (x, y) [43,44]. The intrinsic parameters can be represented in a matrix as:

(1) $$\left[\begin{array}{ccc} f_{\varkappa } & 0 & 0\\ s & f_{y} & 0\\ c_{x} & c_{y} & 1 \end{array}\right]$$

where [$c_{x}{\;c}_{y}$] is the optical centre in pixels, [$f_{x}{\;f}_{y}$] is the focal length in pixels $\left(\frac{F}{P}\right)$, F represents the focal length in world units (mm), P is the pixel in world units, and s is defined by $f_{x}$ tan α, where α is the angle between the image axes.

However, after applying extrinsic and intrinsic translation, due to the radial distortion, the camera captures the values of the distorted pixels $\left(x_{d}{,\;y}_{d}\right)\;$ instead of the real points (x, y). The distorted relation between the distorted points is represented as:

(2) $$x_{d}=x\left({1+k}_{1}r^{2}{+k}_{1}r^{4\;}{+k}_{1}r^{6}\right)$$

(3) $$y_{d}=y\left({1+k}_{1}r^{2\;}{+k}_{1}r^{4}{+k}_{1}r^{6}\right)$$

where x and y are 2D undistorted pixel coordinates after the application of intrinsic projections, and $r^{2}{=x}^{2\;}{+y}^{2}$ and $k_{1}{,\;k}_{2}{,\;k}_{3}$ are the radial distortion coefficients of the lens.

**Figure 4:**
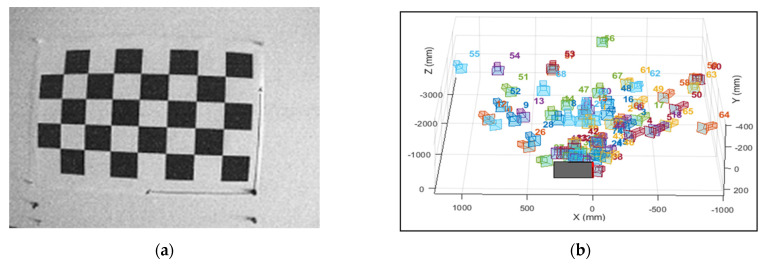
(**a**) A distorted image of a checkerboard pattern from a multispectral camera and (**b**) extrinsic parameters action visualisation. Images were taken from different angles and distances to calculate extrinsic parameters and minimise radial barrel distortion.

This study utilises the traditional checkerboard corner detection method [45] to remove distortion. A 5 × 8 grid checkerboard with a 50 mm size for each square grid was used for this process. For distortion correction, images were taken from different distances and angles using the multispectral camera. The world points were detected from the corners to determine the extrinsic parameters for each band (Figure 4a,b).

#### 2.3.2. Registration of Optical, Multispectral, and Thermal Images

**Figure 5:**
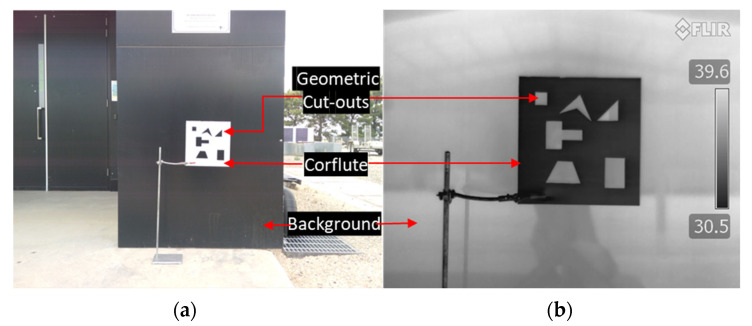
Setup for coarse image registration: (**a**) FoV of RGB; (**b**) FoV of thermal image. A white corflute with different geometric cut-outs was placed in front of a black background with a higher surface temperature than the corflute sheet. The cut-outs are visible in optical (RGB), multispectral, and thermal image bands.

Image registration was used to align multiple images with geometric shifts to create a composite view and improve the signal-to-noise ratio. Image registration matches two or more images acquired from different viewpoints, sensors, time, or FoVs to extract valuable information otherwise impossible from the individual images. The purpose of image registration for this research was to align and stack the images from the multispectral and thermal cameras, which have a different FoV (Figure 5a,b) and sensor plane offsets. The images were aligned for analysis using a two-step image registration process, i.e., a coarse registration followed by a fine registration.

Coarse registration: The coarse registration process involves identifying discernible features using key point descriptors, filtering the image features using a saliency measure such as M-estimator Sample Consensus (MSAC) [46], computing geometric transformation using the filtered image features, and applying the geometric transformation on the image pair for registration [47]. In this implementation process, an image feature-based registration [48] was applied to register optical (RGB), multispectral and thermal images coarsely. Image features are discernible points that are common among two or more images to be aligned.

Typically, checkerboards are used for automated corner detection (using key point descriptors) and matching algorithms (using geometric transformation) to operate on optical input images, e.g., for correct shifts in different spectral bands [11]. However, it is difficult to detect black and white checkerboards in thermal images, as the temperature between the white and black marks remains the same. Thus, a set of geometric shapes cut-out in a corflute sheet (20 × 20 cm) was used to provide discernible and common reference feature points. The geometric shapes cut-out was placed in front of a higher temperature back wall. The setup was arranged to detect geometric corners as image features between the optical, multispectral, and thermal images. The temperature difference can be observed as corners in the geometric shapes in the thermal image, and the difference in colour acts as corners in optical and multispectral images (Figure 5a,b).

The coarse registration step was implemented to provide rigid transformation, which varies with the orientation of the cameras and the distance of imaging. The rigid transformation matrix includes translation and rotation, and nonrigid matrix includes shear and scale with the matrix representations, as shown in Table 1.

**Figure 6:**
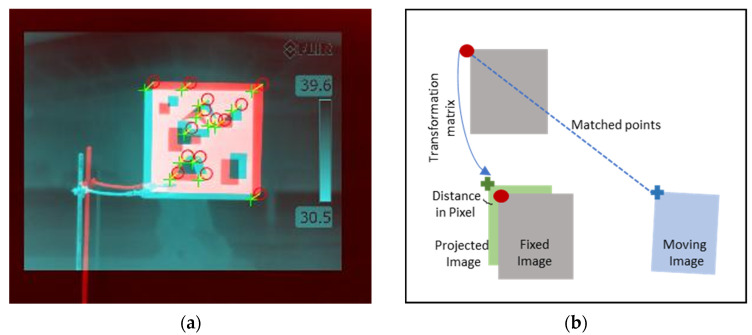
(**a**) Feature detection between RGB and thermal images; the green cross and red circles represent common features (corners) for thermal and multispectral images, respectively. (**b**) Working principle of the projection of moving image into the FoV of a fixed image using a geometric transformations matrix. The red circle, blue cross, and green cross represent the features of the fixed image, moving image, and projected image, respectively.

Translation, scale, and shear measure the displacement, scale factor, and shear along the *x*- and *y*-axis, respectively. The angle of rotation about the origin is denoted by q. These parameters are combined depending on the position and orientation of the two image pairs to create a geometric transformation matrix. Figure 6a shows how a moving image is projected in the FoV of the fixed image. The matched points of fixed and moving image pairs were used to determine the translation, shear, scale, and rotation angle between the two image pairs. These four matrices are concatenated using matrix multiplication to obtain a geometric transformation matrix, which was used to project the moving image into the frame of the fixed image. The coarse registration resulted in fixed and projected image pairs to nearly overlap with minute shifts, represented as distance in pixels (Figure 6b).

**Figure 7:**
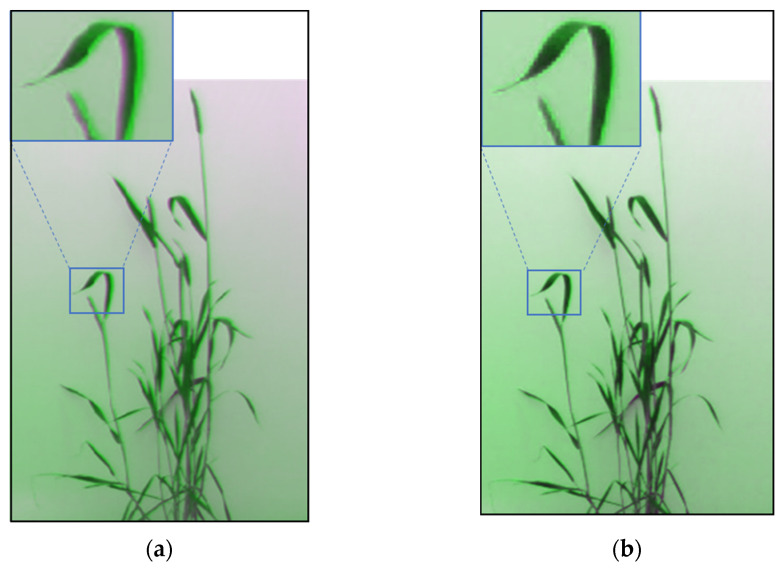
Image registration output after (**a**) coarse registration and (**b**) fine registration. Pink represents misalignment between the thermal and optical images, which was significantly reduced after fine registration.

Fine registration: A fine registration was applied using intensity-based registration [49,50] to fix the misalignment after coarse registration. The misalignment may be caused by the difference in image capture times between the two sensors combined with the movement of the plant’s canopy. Intensity-based registration aligns images based on the pixel intensity levels of the two images that overcome the local anatomical differences. An image similarity metric (Mattes mutual information algorithm) [51] and one-plus-one evolutionary optimiser [52] were used for fine registration. An image similarity metric determines the statistical closeness of pixel-level intensity information between two images, and optimisers work iteratively to minimise the similarity metric, thereby achieving perfect overlap (Figure 7b). The fine registration step involves a nonrigid geometric transformation model involving shear (Sh) and scale (S) transformation in addition to Translation (T) and Rotation (R), as in Table 1. Therefore, the nonrigid transformation model enables an organic alignment of plant tissues such as stems and leaves at fine level, and to a large extent can adjust for minor shaking of plant due to air.

#### 2.3.3. Radiometric Rescaling of Thermal Images

**Figure 8:**
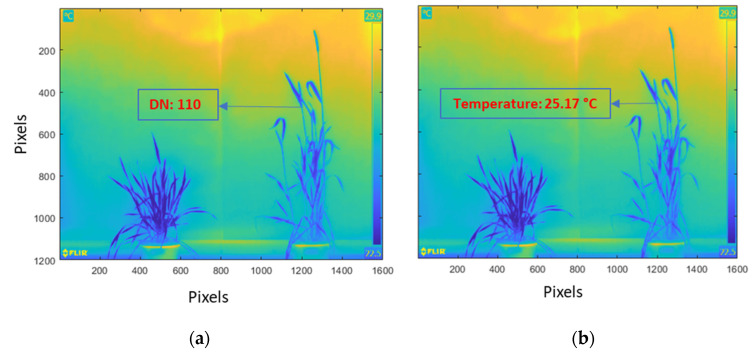
Pixel values of images: (**a**) before radiometric rescaling—the pixel values are stored as Digital Numbers (DNs); (**b**) after radiometric rescaling—the DNs are converted to temperature values. The maximum and minimum temperature values are recorded on the right of the thermal image.

Radiometric rescaling is used to convert the Digital Numbers (DN) to corresponding parametric values. In captured thermal images, temperature values remain scaled as 8-bit DN equivalent in a range of 0–255 (Figure 8a). The first step to convert the DN to temperature values was to extract the maximum (maxT) and minimum temperature (minT), which are, respectively, embedded at the top and bottom of the temperature scale in the captured thermal images. An Optical Character Recognition (OCR) algorithm was used to extract the maxT and minT levels. Secondly, the max and the min DN numbers were determined from each image. Finally, a standard radiometric rescaling model (Equation (4)) was used to convert the DN values to temperature values (Figure 8b).

(4) $$T=\mathrm{minT}+\frac{\mathrm{maxT}-\mathrm{minT}}{\mathrm{maxDN}-\mathrm{minDN}}\times \mathrm{DN}$$

#### 2.3.4. Gradient Removal and Illumination Correction of Multispectral Images

Illumination variation is caused by (i) non-uniformness in the spatial distribution of radiation on the object plane due to the direction of the incident radiation, and (ii) changes in the intensity of the incident radiation through time, i.e., for images taken at different time points [53,54]. Inaccurate retrieval of reflectance images and inaccuracies in image analysis and segmentation, are some of the main problems associated with illumination variation [55].

Traditional ways of correcting illumination involve creating an imaging chamber with perfect light conditions, which is expensive, time-consuming, and not feasible in all glasshouse settings. The method used in this research is suitable for a glasshouse environment, easy to replicate, and cost-effective. This method was carried out in two steps—gradient variance was carried out followed by the illumination correction. A variation in gradient is the change in colour or intensity of images in a certain direction. This is caused due to directional light source during image acquisition and can produce a significant variation in pixel values.

To correct the gradient in the image, first, an interpolated gradient reference image ($G_{\mathrm{ref}}$) was created. A 4 × 4 pixels size Region of Interest (ROI) was selected from the four corners of the original image. Next, the average illumination of these four corners was calculated to interpolate $G_{\mathrm{ref}}\;$ [54]. The difference between $G_{\mathrm{ref}}$ and the minimum pixel value of $G_{\mathrm{ref}}$ was calculated as $G_{\mathrm{dif}}$ (Equation (5)). Finally, the corrected gradient ($G_{\mathrm{cor}}$) was determined by subtracting the original image $\left(I_{\mathrm{org}}\right)$ with the $G_{\mathrm{dif}\;}$(Equation (6)).

(5) $$G_{\mathrm{dif}}{=G}_{\mathrm{ref}\;}-{\min(G}_{\mathrm{ref}})$$

(6) $$G_{\mathrm{cor}}{=I}_{\mathrm{org}}-{\;G}_{\mathrm{dif}}\;$$

**Figure 9:**
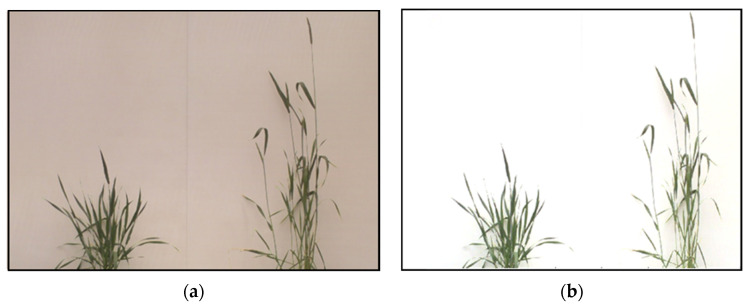
Output of RGB images after (**a**) gradient correction and (**b**) illumination correction.

After the gradient correction, the temporal variation in illumination levels was applied to the corrected image. A dark reference image ($I_{\mathrm{dark}}$) was taken by covering the lenses of the multispectral camera. An interpolated image was created from the four corners of the gradient-corrected image ($G_{\mathrm{cor}}$) as the white reference image ($I_{\mathrm{white}}$). The final corrected image $I_{\mathrm{cor}}$ was derived from Equation (7), where the corrected data were subtracted from the dark reference data and then divided by the difference of the white and dark reference image, and the output was multiplied by the spectral reflectance factor (ref) of the background band, which was 80%. Figure 9a,b show the results after gradient correction and illumination correction; the output after illumination correction facilitates the segmentation process.

(7) $$SI_{\mathrm{cor}}=\frac{G_{\mathrm{cor}}-I_{\mathrm{dark}}}{I_{\mathrm{white}}+I_{\mathrm{dark}}}\times \mathrm{ref}$$

#### 2.3.5. Segmentation to Separate the Plant from the Background

Segmentation is a crucial precursor in image analysis for plants. Image segmentation helps remove the nonvegetative parts, which improves the extraction of spectral and temperature profiles of different parts of plants, such as leaves, stems, and heads [56,57].

**Figure 10:**
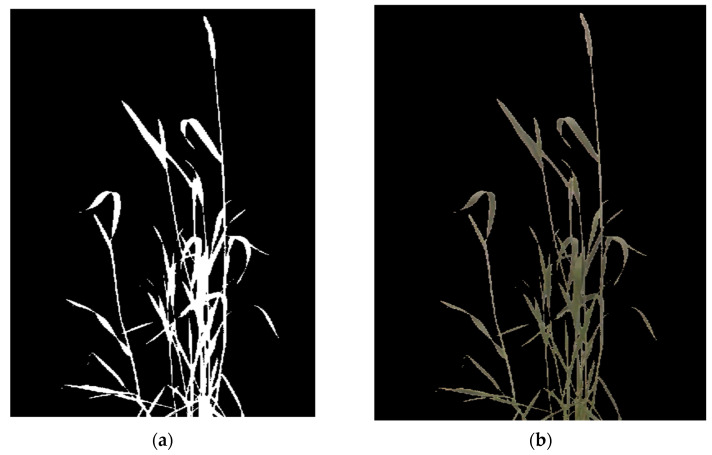
Image segmentation: (**a**) foreground mask after adaptive thresholding and (**b**) segmented RGB image after application of the foreground mask. The non-canopy pixel values are converted to zero.

For this experiment, since the spatial resolution of the RGB was better than the thermal image, the RGB image was used to create a foreground mask for the plant. A segmentation mask was used to extract the plant from the background for thermal and multispectral images [58]. The image segmentation was carried out using an adaptive thresholding method. The adaptive thresholding method has an advantage over fixed thresholding as it provides an optimal threshold of pixels based on the intensity of its neighbour pixels. Additionally, adaptive thresholding solves the issue with shadow pixels, which are incorrectly considered parts of a plant during segmentation in a global thresholding approach [59]. Figure 10a shows the foreground mask and the segmented RGB image and Figure 10b the output of the segmented image.

**Figure 11:**
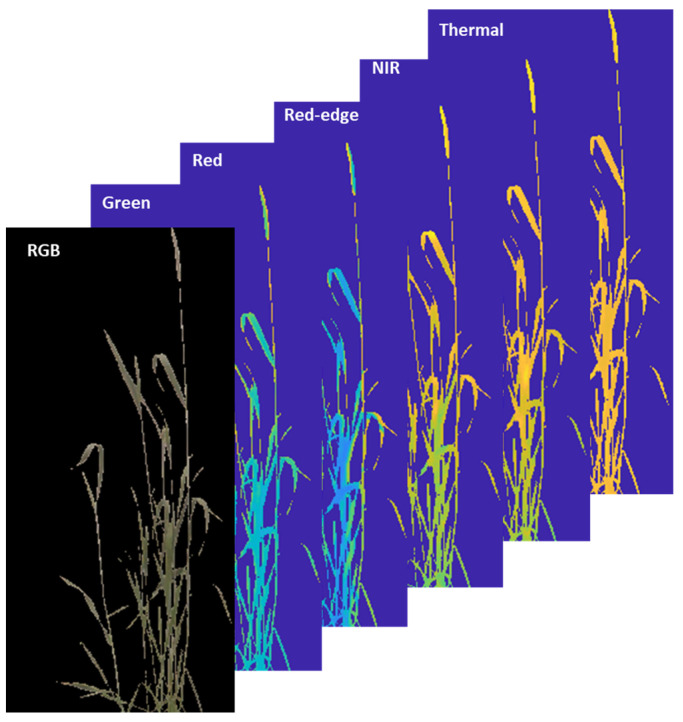
An eight-band stacked image representing only the canopy pixels in the following order: RGB, green, red, NIR, red-edge, and thermal. Each pixel of an image represents the same pixels for the other images, which are represented by red squares in the images.

The image processing pipeline’s output was an eight-band stacked image (Figure 11). The band was stacked in the sequence RGB, green, red, NIR, red-edge, and thermal image. Each of the stacked images only represents the pixel associated with the canopy of the plant, and the remaining pixels were set to Not a Number (NAN), which represents undefined pixel values. The final eight-band image facilitates analysis and comparison of the plant canopies for different biotic and abiotic stresses at different levels simultaneously.

#### 2.3.6. Vegetation Indices

**Figure 12:**
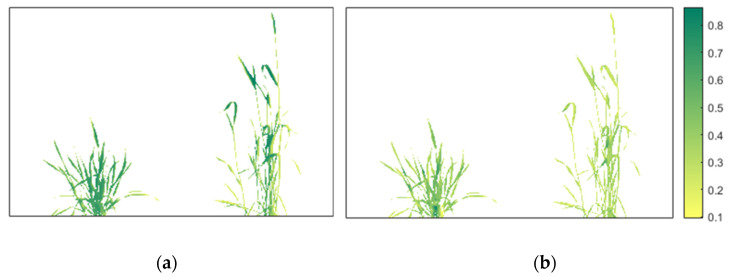
Vegetative indices: (**a**) Normalized Difference VI (NDVI) and (**b**) Chlorophyll Index red edge (CIre).

Vegetation indices were calculated from the eight-band stacked image by using a mathematical combination of several spectral bands to maximise the information obtained from the vegetation while minimising the noise caused by atmospheric effects or reflectance from soil [60]. Biotic and abiotic stresses can highly affect the biophysical property of plants and are correlated to the VIs of crops [61]. NVDI is one of the commonly used indices that combines reflectance from NIR and red light; a low NVDI value indicates the presence of stress in plants caused by biotic or abiotic factors [62,63]. The value of NDVI ranges from −1 to 1, where the higher value represents healthy and dense vegetation [64] (Figure 12a). CI red edge (CIre) is calculated with the help of NIR and red-edge wavelength (Figure 12b). Observing small variations in chlorophyll contents is useful since a linear relationship exists between the reflectance of NIR and the inverse of the red-edge band [65]. Some other VIs that can be associated with the presence of pathogens are related to water content in plants and chlorophyll pigmentation. The Triangle Vegetation Index (TVI) [66] that determines the radiant energy absorption of chlorophyll has been used to classify healthy and unhealthy crops. NDRE is calculated similarly to the NDVI, but uses a red edge instead of the red band [67]. NDRE is used to identify healthy plants during the mid to late stages of plant growth. The modified indices with red edges improve the vegetative indices since red-edge light is highly sensitive to mid- and high-level chlorophyll contents [30]. The package generates the VIs listed in Table 2; however, any VIs having components of green, red, red-edge, NIR wavelengths can be generated by the users.

## 3. Results

The results of the study are split into three subsections. The results show the performance of the three main methods used in the image processing pipeline, i.e., radial distortion correction, image registration, and segmentation.

### 3.1. Correction of Radial Optical Distortion

**Figure 13:**
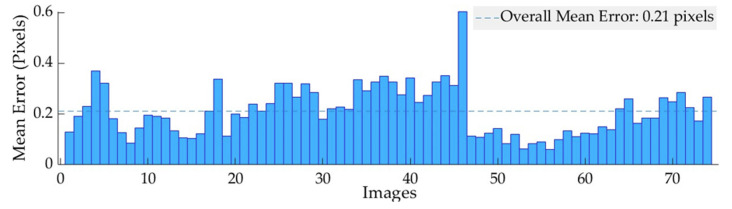
Reprojection error between the distorted and undistorted image for NIR band. The *x*- and *y*-axis represent the number of images and mean errors in pixels, respectively.

The distortion parameters were used to correct the optical distortion of the crop images. The reprojected points (Figure 13) were translated with an overall mean error of 0.21 pixels, with the highest reprojection error of 0.6 pixels for an individual image (NIR band). The reprojection error measures the qualitative accuracy of the undistorted image. The reprojection error is the distance between a pattern key point detected in an undistorted image, and a corresponding reference point projected on the undistorted image. The mean reprojection error for each band is listed in Table 3.

### 3.2. Image Registration

**Figure 14:**
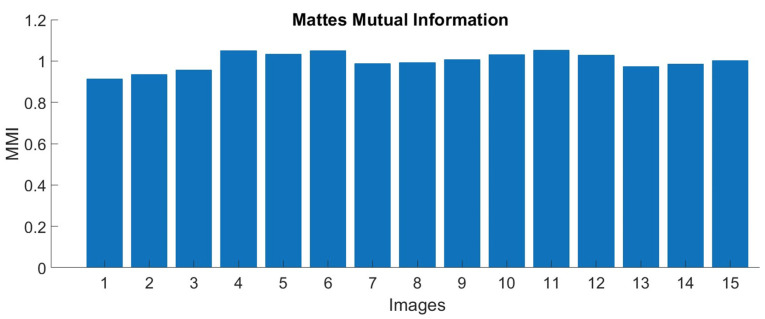
Mattes mutual information between multispectral and thermal images after registration.

Mattes Mutual Information (MMI) [51] was calculated to determine the accuracy of the registration process. The MMI measures how related one set of pixels of an image is to another. The higher MMI implies less entropy among the images and that the images are better aligned. In Figure 14, the bar graph represents MMI, and the image numbers are shown on the *x*-axis. The value of MMI was greater than 0.9, indicating the images were well aligned.

### 3.3. Segmentation

**Figure 15:**
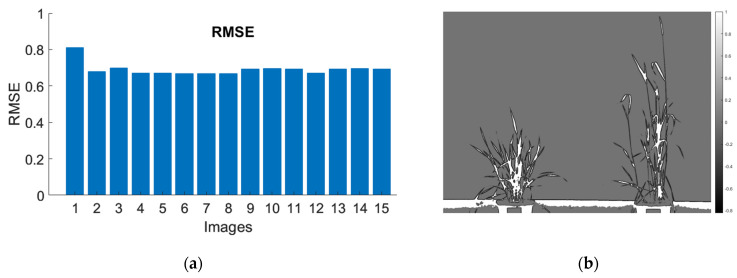
Error metric for segmentation: (**a**) Root Mean Square Error (RMSE) and (**b**) Structural Similarity Map (SSIM) between the original image and segmented images.

Since there was no ground-truth data for the images, Root Mean Square Error (RMSE) and Structural Similarity Map (SSIM) [71] were calculated to find the accuracy of the image segmentation process. Figure 15a represents the RMSE value for images, and the average value of RMSE between the segmented image and the original image was below 0.8.

The SSIM was also used to validate the accuracy of the segmentation process. In the SSIM map, a large local SSIM had bright pixels representing the common regions between two images (Figure 15b).

## 4. Discussion

Digital imaging is pivotal in high-throughput plant phenotyping to characterise morpho-physiological traits reliably and efficiently [1]. For imaging, thermal and multispectral sensors provide essential modalities, i.e., thermal cameras detect the infrared energy emitted from the object to generate a digital image, whereas multispectral cameras convert the light reflected from the object to a visual image. The different modalities of these two cameras can be used in conjunction to add dimensionality to the information [4,5].

An objective of this study was to develop an image processing tool to fuse the information obtained from thermal and multispectral images for application in plant research. For image fusion, special care should be taken so that the two sensors are aligned correctly to provide uniformity in the FoV of the images. Data correction is often one of the most trivial but critical tasks in image processing, and it is essential to correct the different discrepancies in images (distortion, illumination, contrast, etc.) for accurate and swift analysis [72]. This study reports a sequential image processing pipeline to help researchers effectively utilise thermal and multispectral cameras for plant phenotyping. The steps involved in the image processing pipeline include correction of optical distortion, image co-registration, thermal radiometric scaling, background illumination correction, and segmentation.

The radial barrel distortion is a common issue, especially with sensor having smaller focal length [73], which is observed in the green, red, NIR, and red-edge images taken by the multispectral camera [40]. The removal of optical distortion helped solve the distortion so that the corrected images could be used for co-registration and segmentation processes. In this study, a 5 × 8 grid checkerboard pattern with a 50 mm box size was used to calculate the intrinsic and extrinsic parameters of the multispectral camera; the reprojection error of the overall bands was below 0.29 pixels except for the red-edge band (0.6). A similar approach was applied by Das Choudhury, et al. [74] to correct images taken from a Parrot Sequoia camera, using a 28 × 28 mm box size and achieved an average error of less than 0.3 pixels.

Image co-registration was used to fuse the thermal and multispectral images. The idea behind image fusion was to extract information from both cameras. The co-registration of the images was carried out in two steps: feature-based and intensity-based. The feature-based transformation was used to scale and transform the thermal image to an RGB image using a transformation matrix. However, the feature-based transformation is rigid and varies with the distance between the target image and the camera [75]. Hence, the transformation matrix was calculated at variable distances and used for the coarse registration step of the image registration for each imaging setup. To avoid image misalignment caused by the movement of the plant canopy, the imaging was performed in an area of the glasshouse without significant air movement, and image acquisition between thermal and multispectral cameras was closely synchronised.

Radiometric scaling of thermal images was performed as the pixel values are stored as DN values instead of temperature values. Most studies have not explained how the DN values were converted into actual temperature values in thermal images [33]. The only temperature values provided in the thermal image are the minimum and maximum values of temperature recorded for the entire image, which is recorded at the right side of the image. In this study, the maximum and minimum temperature values were extracted with the help of a text recognition algorithm called OCR (Optical Character Recognition) [76], followed by a formula for radiometric scaling to derive each pixel’s temperature value from the DN values. In a recent study, measures were taken to correct the canopy temperature that may be impacted by the emission from the surroundings [33]. However, this approach was not applied in this experiment, since it was carried out in a controlled environment, with the FOV of the thermal camera on the canopy and a fair distance was maintained between the background and the plants. The thermal camera used here captures a radiometric thermogram and saves the file as a radiometric JPG image (RJPG), which allows the adjustment of the distance of object, reflected temperature, emissivity, and surrounding temperature within the camera settings [77].

Segmentation of the background from an object can be challenging due to noise present in the image other than the object of interest [78,79]. A white background was used to help remove background noise to facilitate the segmentation process. Since the intensity and resolution of the RGB image were greater than the thermal image and other bands, the RGB image was used to create a foreground mask that was applied to the remaining images. In a study to identify water stress, Leinonen and Jones [25] have also used visible images to classify vegetative from nonvegetative pixels and extract temperature values of only the vegetation. Adaptive thresholding or local method was utilised for segmentation, this method is useful for nonuniform lightning conditions and solves the problem of shadowing. In another study, the local thresholding method was also utilised to segment the maize canopy from a white background [74].

The image processing pipeline was designed specifically for controlled environment conditions, since, for field conditions, image acquisition has moved from handheld cameras to sensors mounted on unmanned aerial vehicles. Other segmentation methods such as Mask R-CNN [80] and semantic segmentation [81] can be implemented if the setup is to be used in field conditions. Additionally, measures can be applied to correct temperature that may be affected by the thermal emissions and reflections from surrounding objects [33].

Although FLIR thermal cameras provide licensed software for the scaling of thermal images, the users are limited to only temperature values of canopy for their study. A recent study fused RGB and thermal images from a FLIR E60 thermal camera to determine water management in potato [33]. However, this study was only limited to calculation of CWSI from thermal and GRVI from RGB sensors. Researchers are keen on using multiple sensors to study crop phenological changes during stress; however, there is a limitation of open-source packages that helps users to correct and combine information from thermal and multispectral images at pixel level. The output of our study helps to facilitate the researchers with a package that combines the information from thermal and multispectral sensors. This package generates stacked images with eight-bands in the order: RGB (1st–3rd band), green (4th band), red-edge (5th band), NIR (6th band), red (7th band), and thermal (8th band), which contained only the pixels of the plant, which are easy to compare and compute different VIs. Importantly, the image processing pipeline allows batch processing of images to save computational time and efforts. This package enables users to create the already known indices in multispectral bands and create new indices by combining both multispectral and thermal imagery.

## 5. Conclusions

An image processing pipeline was established and packaged to analyse multispectral and thermal images captured in a glasshouse environment. The automated image processing pipeline fixes issues of radial distortion in multispectral images, co-registration of the thermal and multispectral images, normalisation of variation in illumination across the multispectral image, and classification of canopy pixels from background noise. The final output received from the pipeline is a stacked image with an eight-band composite retaining only the canopy pixels for each band, which can be used to create vegetative indices. The process is efficient as images are processed and analysed in batches across all bands. The image processing pipeline will be helpful for researchers working with thermal and multispectral imaging in glasshouse conditions.

## Figures and Tables

**Table 1:** Rigid and nonrigid transformation matrix.

Rigid	Nonrigid
$\mathrm{Translation}\;(T)=\left[\begin{array}{ccc} 1 & 0 & 0\\ 0 & 1 & 0\\ t_{x} & t_{y} & 1 \end{array}\right]$	$\mathrm{Shear}\;\left(\mathrm{Sh}\right)=\left[\begin{array}{ccc} 1 & {\mathrm{sh}}_{y} & 0\\ {\mathrm{sh}}_{\varkappa } & 1 & 0\\ 0 & 0 & 1 \end{array}\right]$
$\mathrm{Rotation}\;\left(R\right)=\left[\begin{array}{ccc} \cos(q) & \sin(q) & 0\\ -\sin(q) & \cos(q) & 0\\ 0 & 0 & 1 \end{array}\right]$	$\mathrm{Scale}\;\left(S\right)=\left[\begin{array}{ccc} s_{\varkappa } & 0 & 0\\ 0 & s_{y} & 0\\ 0 & 0 & 1 \end{array}\right]$

**Table 2:** Vegetation indices generated from the image processing pipelines.

Indices	Equations	References
Normalized Difference Vegetation Index (NDVI)	$\mathrm{NDVI}=\frac{\mathrm{NIR}\;-\;\mathrm{RED}}{\mathrm{NIR}\;+\;\mathrm{RED}}$	[8]
Normalized Difference Red Edge (NDRE)	$\mathrm{NDRE}=\frac{\mathrm{NIR}\;-\;\mathrm{RED}\_\mathrm{EDGE}}{\mathrm{NIR}\;+\;\mathrm{RED}\_\mathrm{EDGE}}$	[12]
Chlorophyll Index red edge (CIre)	$\mathrm{CIre}=\frac{\mathrm{NIR}}{\mathrm{RED}\_\mathrm{EDGE}}-1$	[14]
Triangle Vegetation Index (TVI)	0.5(120($\mathrm{NIR}\;-\;\mathrm{GREEN})-200\left(\mathrm{RED}\;-\;\mathrm{GREEN}\right))$	[66]
Renormalized Difference Vegetation Index (RDVI)	$\mathrm{RDVI}=\frac{\mathrm{NIR}\;-\;\mathrm{RED}}{\sqrt{\mathrm{NIR}\;+\;\mathrm{RED}}}$	[68]
Chlorophyll Vegetation Index (CVI)	$\mathrm{CVI}=\mathrm{NIR}\;\frac{\mathrm{RED}}{{\mathrm{GREEN}}^{2}}$	[69]
Chlorophyll Index green (CIg)	$\mathrm{CIg}=\frac{\mathrm{NIR}}{\mathrm{GREEN}}-1$	[70]

**Table 3:** Mean reprojection error between the distorted and undistorted image for different multispectral bands.

Bands	Red	Red-Edge	NIR	Green
Mean reprojection error (pixels)	0.29	0.60	0.21	0.20

## Data Availability

The data is freely shared in google drive and can be accessed from the following link. https://drive.google.com/file/d/1VSqRu5CUZhyd3MF23kdRjqrtRke7sbJU/view?usp=share_link.
